# A serpent-type wave energy converter

**DOI:** 10.1038/s41598-023-39337-6

**Published:** 2023-08-01

**Authors:** Sebastian Sorek, Wojciech Sulisz

**Affiliations:** Department of Wave Mechanics and Structural Dynamics, Institute of Hydro-Engineering PAS, Kościerska 7, 80-328 Gdańsk, Poland

**Keywords:** Civil engineering, Devices for energy harvesting, Hydroelectricity

## Abstract

The problem of the interaction of waves with a serpent-type wave energy converter was investigated, and a novel 3D analytical solution was derived. The optimal parameters of the energy converter were derived for the first time from wave energy principles. The results show that the wave power captured by the device increases with increasing wave lengths until a maximum and then decreases. The efficiency increases with decreasing stiffness of the device in shallow and intermediate waters. In deep water, the efficiency increases with increasing stiffness until a local maximum and then decreases. Moreover, the efficiency increases with the increasing mass of the device in shallow and intermediate waters. In deep water, the efficiency of a converter decreases with the increasing mass of the device. An original approach to determine the optimal parameters of a device for given wave conditions was derived. The derived analytical formula shows that the top efficiency level of power capturing cannot exceed 50%. This is the theoretical maximum for this type of converter. The power take-off optimization analysis also identifies the spectrum of wave conditions for which the efficiency of the generator is close to the maximum. A series of laboratory experiments were conducted in a hydraulic laboratory to verify the model. The comparisons of the recorded data with the analytical solution show a very good agreement.

## Introduction

Potential energy of ocean waves is estimated to be about 2.96 TW^1^. It is a substantial resource that is not being utilized into electrical energy. The perspective of rising global energy consumption and the unwanted side-effects of fossil fuels energy production brings even more importance to the issue of acquiring wave energy. Despite the current low level of conversion, the studies regarding this topic and propositions of mechanisms for capturing this resource are quite extensive. This suggests that the optimal technology is yet to be determined and potential for advancements and innovations in this field exists.

First investigations into the possibility of utilizing wave energy were conducted by Stahl^2^ more than a century ago. A serious revoked interest in the subject came in the seventies^3^ in the midst of the international oil crisis when a promising wave oscillating mechanism was presented by Salter in 1974^4^. These circumstances brought more funding and scientific interest to the topic of wave energy utilization. After not identifying a viable, affordable solution for wave energy conversion, the interest and investments declined around 1984. Only in the last twenty years, with the growing concern of climate change the technology have again gained more attention. Correspondingly, a surge in research and development of new devices has ensued, especially after the commercial success of other renewable energy technologies, e.g. wind turbines.

A variety of new proposed designs and prototypes led to attempts to categorize and organize wave energy converters (WECs). Different types of classifications emerged. Oscillating water column (OWC), overtopping devices, diaphragm pressure differential devices and oscillating body devices are groups that sort converters depending on their principle of capturing wave energy.

Oscillating water columns work by having an air chamber above water from which wave oscillations are pushing air in and out through a turbine. An example of an OWC device is described by Gish^5^. Overtopping devices collect water from overtopping waves to a tank from which the potential energy of that water is utilized. Please see the example of a working device presented by Margheritini et al.^6^. Pressure differential devices are submerged constructions that use the changing pressure, due to the wave’s motion, above them to pump fluid inside the devices. A large class of devices, the oscillating body converters, extract energy from waves induced oscillations of parts of their bodies. Pelamis^7^ and Oyster^8^ are more commonly known oscillating body converters.

Another main classification separates converters by the type of mode of motion of the working surface of devices relative to the water surface. For the most efficient wave energy extraction, the working surface of a wave energy converter moves in the vertical heave motion^9^, the horizontal surge motion^10^, the tilting pitch motion^11^, or some combination of those.

Devices can still be more precisely categorized by specifying the type of location where they operate: offshore, near-shore and shoreline; or their orientation regarding the incoming waves: terminators, attenuators and point absorbers. A more complete description of current technological classifications is provided by Drew et al.^12^, and for a general review of various working wave energy converters, see Joubert et al.^13^.

Despite various proposed devices, the main problem concerning the implementation of this technology remains the same. The Levelized Cost of Energy (LCOE) from the wave energy conversion exceeds several times that of other, more developed renewable technologies^14^. This is most commonly attributed to the high cost of maintenance and the cost of the device itself^15^. One solution is to develop an optimal WEC design for effective and reliable energy production, so it can be easily reproduced and scaled. Another arising idea regarding solving this issue is to justify the cost by adding additional functions to devices or using them in highly specific settings: combining breakwater and coastal protection with wave energy conversion^16,17^, combining wave energy devices with wind energy devices^18^, powering desalination plants^19^, fishing farms or offshore rigs^20^.

In this study, a new concept of an efficient 3D WEC that is capable of subtracting energy from the entire water column is introduced and investigated. The device is a terminator, an oscillating body-type WEC, which captures energy from the wave motion. The converter is planned as a row of independently moving plates that oscillate in the horizontal direction. It is intended to operate near-shore with plates standing from the sea bottom to the sea surface. Wave energy is retrieved by the mechanism of capturing and converting the mechanical energy of the oscillating plates. The proposed concept is original and substantially differs from a single porous-plate WEC installed at a seawall (Ching-Yun and Shih-Hsuan^21^) or partially-submerged plates installed on a floating tube perpendicular to the shoreline (Angelelli et al.^22^).

Additionally, the WEC may be applied as a shore protection device. The proposed converter is intended to cover long coastline distances, and capturing part of wave energy reduces wave’s intensity and can be used for shore protection. In fact, Evans and Linton^23^ analyzed this aspect of wave energy converters. Millar et al.^24^ and Mendoza et al.^25^ considered possibilities to use this mechanism in practice. A review of prototype devices is found in Mustapa et al.^26^.

For the described WEC, first a boundary-value problem is formulated to describe the wave interaction with the serpent-type wave energy converter. Then, the analytical solution for the proposed wave energy converter is derived. The model is applied to predict the interaction of the device with water waves. The results are discussed with emphasis on the wave load on the converter and the optimal parameters of the wave energy converter. The method of experimental verification of the theoretical solution is provided and explained in the Experimental verification section. Finally, an analysis of the effect of the parameters on the energy collection is conducted, and conclusions are formulated in the final section.

## Theory

### Governing equations and solution

**Figure 1:**
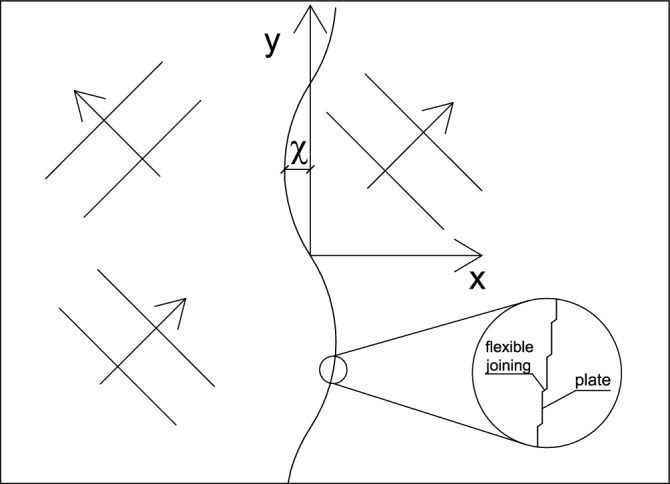
Sketch of the serpent-type wave energy converter with the coordinate system.

The situation considered in the study is the wave interaction with a device constructed as a row of plates, which can move horizontally in piston-type oscillations. The plates are connected by flexible-type joinings. The situation is shown schematically in Fig. 1, where χ (*y, z, t*) is the plate displacement.

The solution for the wave-device interactions is derived on the basis of the potential wave theory, which is a standard theory used in the modelling of wave energy converters. This approach has some limitations and requires the following assumptions. The fluid is inviscid and incompressible, and the fluid motion is irrotational. Moreover, it is assumed that the sea bottom is impervious, and that the excitation is provided by incident waves of small amplitude *A* and frequency ω. These are reasonable and widely recognized approximations applied in the analyses of real working WECs.

According to these assumptions, the velocity vector, ***V*** (*x, y, z, t*), may be computed from the velocity potential Φ (*x, y, z, t*)

1 $${\varvec{V}}=\mathrm{\Delta \Phi }\left(x,y,z,t\right),$$

where ∇ (^.^) is the two-dimensional vector differential operator

The fluid motion is governed by the continuity equation

2 $${\nabla }^{2}\Phi =0,$$

The kinematic boundary condition,

3 $${\upeta }_{\mathrm{t}}+{\Phi }_{x}{\upeta }_{\mathrm{x}}+{\Phi }_{\mathrm{y}}{\upeta }_{\mathrm{y}}-{\Phi }_{\mathrm{z}}=0, z=\upeta \left(x,y,t\right).$$

The dynamic boundary condition,

4 $${\Phi }_{\mathrm{t}}+g\upeta +\frac{1}{2}{|\nabla\Phi |}^{2}=0, z=\upeta \left(x,y,t\right).$$

The kinematic boundary at the wave energy converter,

5 $${\upchi }_{\mathrm{t}}+{\Phi }_{\mathrm{y}}{\upchi }_{\mathrm{y}}+{\Phi }_{\mathrm{z}}{\upchi }_{\mathrm{z}}-{\Phi }_{\mathrm{x}}=0, x =\upchi \left(y,z,t\right) ,$$

and at the sea bottom, the following boundary condition must be satisfied,

6 $${\Phi }_{\mathrm{z}} = 0, z = -h ,$$

where η (*x, y, t*) is the free surface oscillation, *g* = 9.81 m/s^2^ is the acceleration due to gravity, and* h* is the water depth.

Furthermore, the velocity potential must satisfy the boundary conditions at infinity and initial conditions^27,28^.

To obtain an analytical solution of the boundary-value problem and to avoid problems arising from an unknown location of the free surface η *(x,y,t),* the Taylor series expansion technique and the perturbation method are applied. The free-surface kinematic and dynamic boundary conditions and the kinematic boundary condition at the wave energy converter were expanded into Taylor series about a mean position^29^.

7 $$\sum_{n=0}^{\infty }\frac{{\upeta }^{n}}{n!}\frac{{\partial }^{n}}{\partial {z}^{n}}({\upeta }_{\mathrm{t}}+{\Phi }_{\mathrm{x}}{\upeta }_{\mathrm{x}}+{\Phi }_{\mathrm{y}}{\upeta }_{\mathrm{y}}-{\Phi }_{\mathrm{z}})=0, z=0,$$

8 $$\sum_{n=0}^{\infty }\frac{{\upeta }^{n}}{n!}\frac{{\partial }^{n}}{\partial {z}^{n}}({\Phi }_{\mathrm{t}}+g\upeta +\frac{1}{2}{\left|\nabla\Phi \right|}^{2})=0, z=0$$

9 $$\sum_{n=0}^{\infty }\frac{{\upchi }^{n}}{n!}\frac{{\partial }^{n}}{\partial {x}^{n}}({\upchi }_{\mathrm{t}}+{\Phi }_{\mathrm{y}}{\upchi }_{\mathrm{y}}+{\Phi }_{\mathrm{z}}{\upchi }_{\mathrm{z}}-{\Phi }_{\mathrm{x}})=0, x=0$$

The application of Eq. (2) to (1) results in following

10 $${\nabla }^{2}\Phi =0,$$

11 $${\Phi }_{\mathrm{t}}+g\upeta +\frac{1}{2}{|\nabla\Phi |}^{2}=0, z=0 ,$$

12 $${\upeta }_{\mathrm{t}}-{\Phi }_{z}-\upeta {\Phi }_{zz}+{\Phi }_{\mathrm{x}}{\upeta }_{\mathrm{x}}+{\Phi }_{\mathrm{y}}{\upeta }_{\mathrm{y}}=0, z=0$$

13 $${\upchi }_{\mathrm{t}}+{\Phi }_{\mathrm{y}}{\upchi }_{\mathrm{y}}+{\Phi }_{\mathrm{z}}{\upchi }_{\mathrm{z}}-{\Phi }_{\mathrm{x}}-{\mathrm{\chi \Phi }}_{\mathrm{xx}}=0, x =0$$

14 $${\Phi }_{\mathrm{z}}=0, z=-\mathrm{h}$$

By assuming perturbation procedure

15 $$\Phi ={}_{1}\Phi +{}_{2}\Phi + ... ,$$

16 $$\upeta ={}_{1}\upeta +{}_{2}\upeta + ... ,$$

where a quantity with a left subscript *n, n* = 0, 1,… is of order of *(Ak)*^*n*^ in which *A* is the characteristic wave amplitude, *k* is the characteristic wave number and *Ak* = ε ≤ 1.

All the following analysis will regard the first order solution of the perturbation method and for the purpose of clarity, the left subscript will be left out.

To further break down the solution, the spatial velocity potential is introduced, such that

17 $$\Phi =\mathrm{Re}\left[\phi {\mathrm{e}}^{-i\upomega t}\right],$$

where ϕ(*x,y,z*) is the spatial velocity potential.

The incident ϕ_I_, reflected ϕ_1_ and transmitted ϕ_2_ spatial velocity potentials may be written in the following form

18 $${\phi }_{I}=\frac{-igA}{\upomega }\frac{\mathrm{cos}{\alpha }_{1}\left(z+h\right)}{\mathrm{cos}{\mathrm{\alpha }}_{1}h}\mathrm{exp}\left(-{\mathrm{\alpha }}_{1}\mathrm{cos\theta }x-{\mathrm{\alpha }}_{1}sin\uptheta y\right),$$

.

19 $${\phi }_{1}={\phi }_{I}+\sum_{j=1}\frac{-ig{R}_{j}}{\upomega }\frac{\mathrm{cos}{\alpha }_{j}\left(z+h\right)}{\mathrm{cos}{\mathrm{\alpha }}_{\mathrm{j}}h}\mathrm{exp}\left(-{\upbeta }_{\mathrm{j}}x-{\mathrm{\alpha }}_{1}sin\uptheta y\right),$$

20 $${\phi }_{2}=\sum_{j=1}\frac{-ig{T}_{j}}{\upomega }\frac{\mathrm{cos}{\alpha }_{j}\left(z+h\right)}{\mathrm{cos}{\mathrm{\alpha }}_{\mathrm{j}}h}\mathrm{exp}\left(-{\upbeta }_{\mathrm{j}}x-{\mathrm{\alpha }}_{1}sin\uptheta y\right),$$

provided that

21 $$\frac{{\omega }^{2}}{g}=-{\mathrm{\alpha }}_{\mathrm{j}}\mathrm{tan}{\mathrm{\alpha }}_{\mathrm{j}}h,j\ge 1$$

22 $${\upbeta }_{\mathrm{j}}=\sqrt{{\mathrm{\alpha }}_{\mathrm{j}}^{2}-{\mathrm{\alpha }}_{1}^{2}{\mathrm{sin}}^{2}\uptheta },$$

where θ is the wave propagation angle and the eigenvalues are: α_j_ = {– *ik*, α_2_, α_3_, …}, where *k* is the wavenumber.

The coefficients *R*_*j*_ can be derived by multiplying the wave energy converter’s kinematic boundary condition (13) by orthogonal functions, cos α_j_ = {cos α_1_, cos α_2_, …}, and then integrating the product, so that the solution for each *R*_*j*_ is

23 $${\upchi }_{10}{\mathrm{sin}}^{2}{\mathrm{\alpha }}_{\mathrm{j}}h={\updelta }_{1\mathrm{j}}{A}_{1}{\mathrm{\alpha }}_{1}\mathrm{cos\theta }\frac{2{\mathrm{\alpha }}_{1}h+\mathrm{sin}2{\mathrm{\alpha }}_{1}h}{4{\mathrm{\alpha }}_{1}}-{\upbeta }_{\mathrm{j}}{R}_{j}\frac{2{\mathrm{\alpha }}_{\mathrm{j}}h+\mathrm{sin}2{\mathrm{\alpha }}_{\mathrm{j}}h}{4{\mathrm{\alpha }}_{\mathrm{j}}},$$

24 $${{R}_{j}=\updelta }_{1\mathrm{j}}{A}_{1}{\mathrm{\alpha }}_{1}\frac{\mathrm{cos\theta }}{{\upbeta }_{1}}-4{\upchi }_{10}{\mathrm{\alpha }}_{\mathrm{j}}\frac{{\mathrm{sin}}^{2}{\mathrm{\alpha }}_{\mathrm{j}}h}{{\upbeta }_{\mathrm{j}}\left(2{\mathrm{\alpha }}_{\mathrm{j}}h+\mathrm{sin}2{\mathrm{\alpha }}_{\mathrm{j}}h\right)},$$

A similar procedure is applied to obtain the coefficients *T*_*j*_

25 $$i\upomega {\upchi }_{10}=-\frac{ig}{\upomega }\sum {\upbeta }_{\mathrm{j}}{T}_{j}\frac{\mathrm{cos}{\mathrm{\alpha }}_{\mathrm{j}}\left(z+h\right)}{\mathrm{cos}{\mathrm{\alpha }}_{\mathrm{j}}h},$$

26 $${\upchi }_{10}{sin}^{2}{\mathrm{\alpha }}_{\mathrm{j}}h={\upbeta }_{\mathrm{j}}{T}_{j}\frac{2{\mathrm{\alpha }}_{\mathrm{j}}h+\mathrm{sin}2{\mathrm{\alpha }}_{\mathrm{j}}h}{4{\mathrm{\alpha }}_{\mathrm{j}}},$$

27 $${T}_{j}=\frac{{4\upchi }_{10}{{\mathrm{\alpha }}_{\mathrm{j}}sin}^{2}{\mathrm{\alpha }}_{\mathrm{j}}h}{{\upbeta }_{\mathrm{j}}\left(2{\mathrm{\alpha }}_{\mathrm{j}}h+\mathrm{sin}2{\mathrm{\alpha }}_{\mathrm{j}}h\right)},$$

where

28 $${\upchi }_{1}={\upchi }_{10}{\mathrm{e}}^{-i\upomega t}.$$

The generalized hydrodynamic forces $F$ for a row of wave energy converters can be calculated at a given location by integrating the wave pressure *p* that is acting on the wave energy converter. Accordingly, where ***n*** is the generalized normal vector.

29 $${\varvec{F}}={\int }_{-h}^{0}p{\varvec{n}} dz,$$

30 $$p=i\mathrm{\omega \rho }\phi {\mathrm{e}}^{-i\upomega t}.$$

The complex-valued amplitude of a horizontal force is

31 $${F}_{10}=\uprho g\left(A\frac{\mathrm{tan}{\mathrm{\alpha }}_{1}h}{{\mathrm{\alpha }}_{1}}+\sum_{j=1}{\mathrm{R}}_{\mathrm{j}}\frac{\mathrm{tan}{\mathrm{\alpha }}_{j}h}{{\mathrm{\alpha }}_{j}}-\sum_{j=1}{\mathrm{T}}_{\mathrm{j}}\frac{\mathrm{tan}{\mathrm{\alpha }}_{j}h}{{\mathrm{\alpha }}_{j}}\right)$$

Analogously, the spatial momentum affecting the converter with respect to the sea bottom

32 $${F}_{50}=\uprho g\left(A\frac{{\mathrm{\alpha }}_{1}h\mathrm{sin}{\mathrm{\alpha }}_{1}h+\mathrm{cos}{\mathrm{\alpha }}_{1}h-1}{{\mathrm{\alpha }}_{1}^{2}\mathrm{cos}{\mathrm{\alpha }}_{1}h}+\sum_{j=1}{\mathrm{R}}_{\mathrm{j}}\frac{{\mathrm{\alpha }}_{j}h\mathrm{sin}{\mathrm{\alpha }}_{j}h+\mathrm{cos}{\mathrm{\alpha }}_{j}}{{\mathrm{\alpha }}_{j}^{2}\mathrm{cos}{\mathrm{\alpha }}_{j}h}-\sum_{j=1}{\mathrm{T}}_{\mathrm{j}}\frac{{\mathrm{\alpha }}_{j}h\mathrm{sin}{\mathrm{\alpha }}_{j}h+\mathrm{cos}{\mathrm{\alpha }}_{j}}{{\mathrm{\alpha }}_{j}^{2}\mathrm{cos}{\mathrm{\alpha }}_{j}h}\right).$$

### Equation of motion

**Figure 2:**
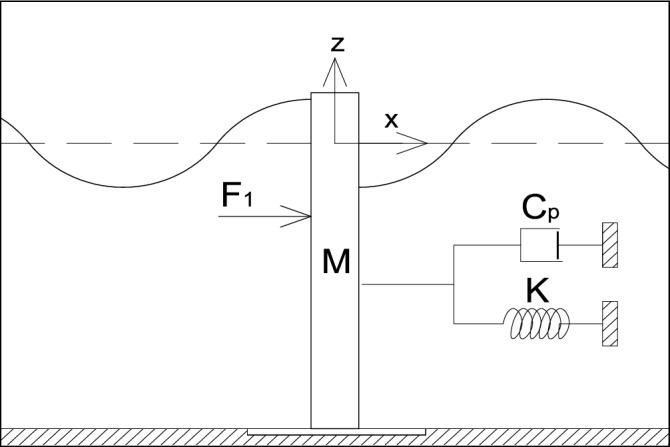
The schematic view power take-off mechanism.

The second part of the analysis establishes and implements to the model the equation of motion describing the effects of the hydrodynamic force on the motion of the wave energy converter (Fig. 2). The equation may be written in the following form

33 $$M{\upchi }_{1}^{{\prime}{\prime}}+{C}_{p}{\upchi }_{1}{\prime}+K{\upchi }_{1}={F}_{1},$$

which may be written as

34 $$-M{\upomega }^{2}{\upchi }_{10}-i{\upomega C}_{p}{\upchi }_{10}+K{\upchi }_{10}={F}_{10},$$

in which *M* – the mass of the structure, *C*_p_– the power take-off coefficient and *K* – the stiffness coefficient.

By providing the force obtained from the Bernoulli equation, the equation of motion may be written as follows

35 $$-M{\upomega }^{2}{\upchi }_{10}-i{\upomega C}_{p}{\upchi }_{10}+K{\upchi }_{10}=\uprho gA\frac{\mathrm{tan}{\mathrm{\alpha }}_{1}h}{{\mathrm{\alpha }}_{1}}+\uprho g\sum_{j=1}{\mathrm{R}}_{\mathrm{j}}\frac{\mathrm{tan}{\mathrm{\alpha }}_{j}h}{{\mathrm{\alpha }}_{j}}-\uprho g\sum_{j=1}{\mathrm{T}}_{\mathrm{j}}\frac{\mathrm{tan}{\mathrm{\alpha }}_{j}h}{{\mathrm{\alpha }}_{j}},$$

which after applying (24) and (27) finally results in an equation for χ_10_ written as follows

36 $${\upchi }_{10}\left(-M{\upomega }^{2}-i{\upomega C}_{p}+K+8\uprho g\sum_{j=1}\frac{{sin}^{2}{\mathrm{\alpha }}_{\mathrm{j}}h}{{\upbeta }_{\mathrm{j}}\left(2{\mathrm{\alpha }}_{\mathrm{j}}h+\mathrm{sin}2{\mathrm{\alpha }}_{\mathrm{j}}h\right)cos{\mathrm{\alpha }}_{\mathrm{j}}h}\right)=2\uprho gA\frac{\mathrm{tan}{\mathrm{\alpha }}_{1}h}{{\mathrm{\alpha }}_{1}}.$$

The Eq. (36) may be written as follows

37 $${\upchi }_{10}\left(-{\upomega }^{2}\left(M+\mu \right)+K-i\upomega \left({C}_{p}+C\right)\right)={F}_{D10},$$

in which we define μ as added mass and *C* as added damping

38 $$\mu =\sum_{j=1}8\uprho \frac{{sin}^{2}{\mathrm{\alpha }}_{\mathrm{j}}h}{{{\mathrm{\alpha }}_{\mathrm{j}}\upbeta }_{\mathrm{j}}\left(2{\mathrm{\alpha }}_{\mathrm{j}}h+\mathrm{sin}2{\mathrm{\alpha }}_{\mathrm{j}}h\right)},$$

39 $$C=8\mathrm{\rho \omega }\frac{{sin}^{2}{\mathrm{\alpha }}_{1}h}{{{i\mathrm{\alpha }}_{1}\upbeta }_{1}\left(2{\mathrm{\alpha }}_{1}h+\mathrm{sin}2{\mathrm{\alpha }}_{1}h\right)},$$

and *F*_*D10*_ as the force due to diffracted waves

40 $${F}_{D10}=2\uprho gA\frac{\mathrm{tan}{\mathrm{\alpha }}_{1}h}{{\mathrm{\alpha }}_{1}},$$

The motion of a wave energy converter depends on the value of the coefficient *C*_*p*_. The selection of an optimal *C*_*p*_ is a complex problem. For small values of *C*_*p*_, the wave energy radiates from a conversion system in the form of reflected and transmitted waves, and the efficiency of a wave energy converter is low. When the values of *C*_*p*_ are large, the wave energy radiate from a system in the form of reflected waves, and the efficiency of a wave energy converter is again low. These indicate that there is an optimal value of *C*_*p*_. The optimal value of *C*_*p*_ may be derived analytically by determining the minimum of wave energy radiated from a wave energy converter

41 $${|{T}_{1}|}^{2}+{|{R}_{1}|}^{2}={A}^{2}-4A\left({\upchi }_{10}+\overline{{\upchi }_{10}}\right)\left(\frac{{\mathrm{sinh}}^{2}kh}{2kh+\mathrm{sinh}2kh}\right)+32{\upchi }_{10}\overline{{\upchi }_{10}}{\left(\frac{{\mathrm{sinh}}^{2}kh}{2kh+\mathrm{sinh}2kh}\right)}^{2},$$

where $\overline{{\chi }_{10}}$ is the complex conjugate.

After some algebra one obtains

$$-8A{F}_{D10}\upomega \frac{{\upomega }^{2}\left(-{C}^{2}-2C{C}_{p}+{\left(M+\mu \right)}^{2}{\upomega }^{2}-{C}_{p}^{2}\right)+{K}^{2}-2K{\upomega }^{2}\left(M+\mu \right)}{{\left[{\left(K-{\upomega }^{2}\left(M+\mu \right)\right)}^{2}+{\upomega }^{2}{\left(C+{C}_{p}\right)}^{2}\right]}^{2}}\frac{{\mathrm{sinh}}^{2}kh}{2kh+\mathrm{sinh}2kh}$$

42 $$-64{{F}_{D10}}^{2}{\upomega }^{2}\frac{C+{C}_{p}}{{\left[{\left(K-{\upomega }^{2}\left(M+\mu \right)\right)}^{2}+{\upomega }^{2}{\left(C+{C}_{p}\right)}^{2}\right]}^{2}}{\left(\frac{{\mathrm{sinh}}^{2}kh}{2kh+\mathrm{sinh}2kh}\right)}^{2}=0,$$

which results in simplified elegant formula for *C*_*p*_

43 $${C}_{p}=\sqrt{{\left(K/\upomega -\upomega \left(M+\mu \right)\right)}^{2}+{C}^{2}},$$

because

44 $$C=-8\mathrm{\rho \omega }\frac{{sinh}^{2}kh}{{{\mathrm{\alpha }}_{1}}^{2}\left(2kh+\mathrm{sinh}2kh\right)}=4\frac{{F}_{D10}}{A\upomega } \frac{{sinh}^{2}kh}{2kh+\mathrm{sinh}2kh},$$

For further evaluation of a wave energy converter efficiency, the incident wave power *P*_*I*_ is calculated from

45 $${P}_{I}=\frac{1}{4} \frac{\uprho g{A}^{2}\upomega }{k}\left(1+\frac{2kh}{\mathrm{sinh}2kh} \right),$$

the power captured *P*_*c*_ by the converter is

46 $${P}_{c}=\frac{1}{2} {C}_{p}{\upomega }^{2}\frac{{\left|{F}_{D10}\right|}^{2}}{{\left(K-{\upomega }^{2}\left(M+\mu \right)\right)}^{2}+{\left(\upomega \left(C+{C}_{p}\right)\right)}^{2}},$$

which may be further simplified to

47 $${P}_{c}=\frac{{\left|{F}_{D10}\right|}^{2}}{4{C}_{p}+4C}.$$

## Results

**Figure 3:**
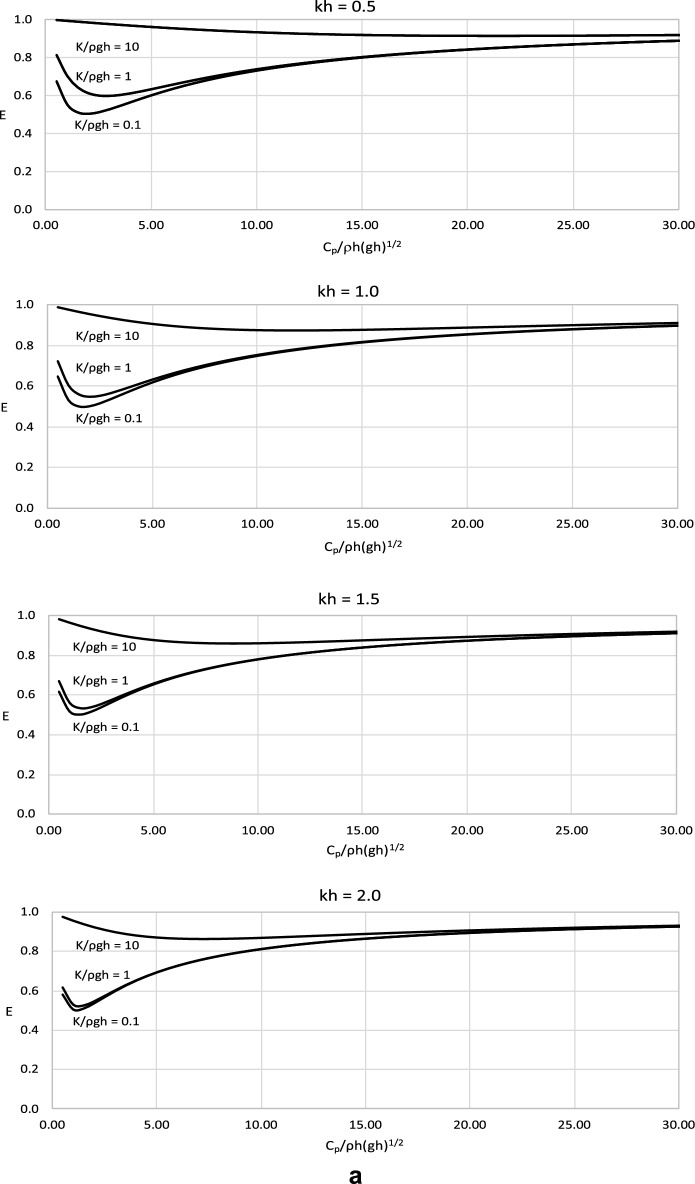

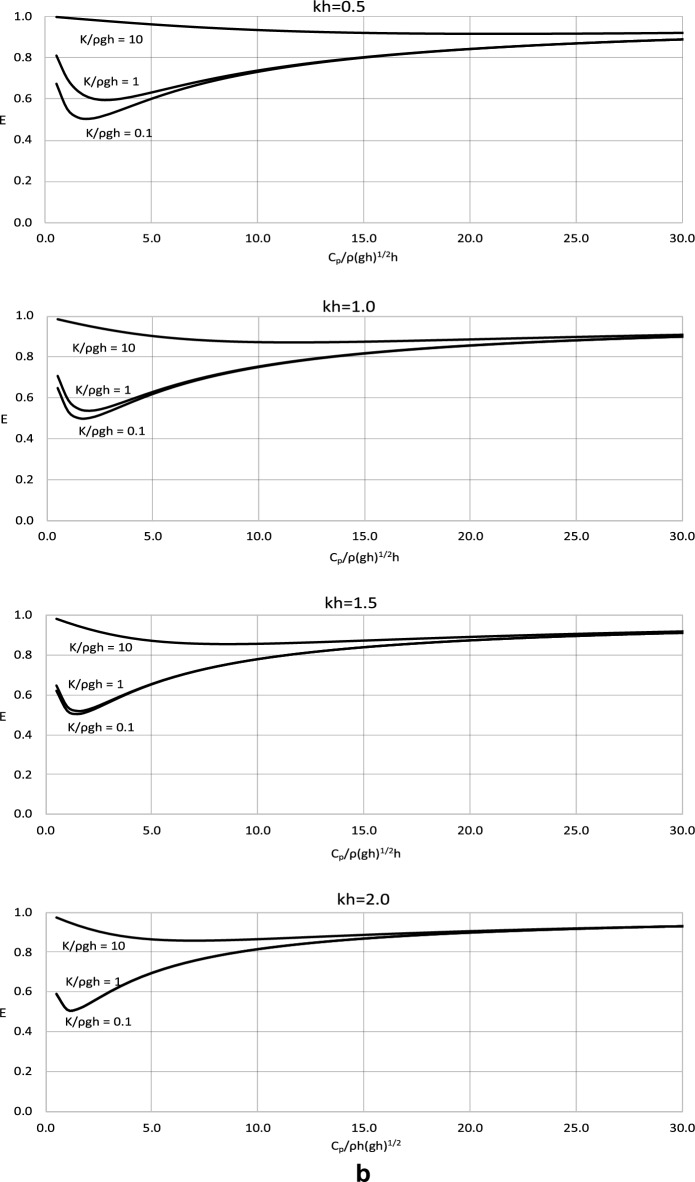

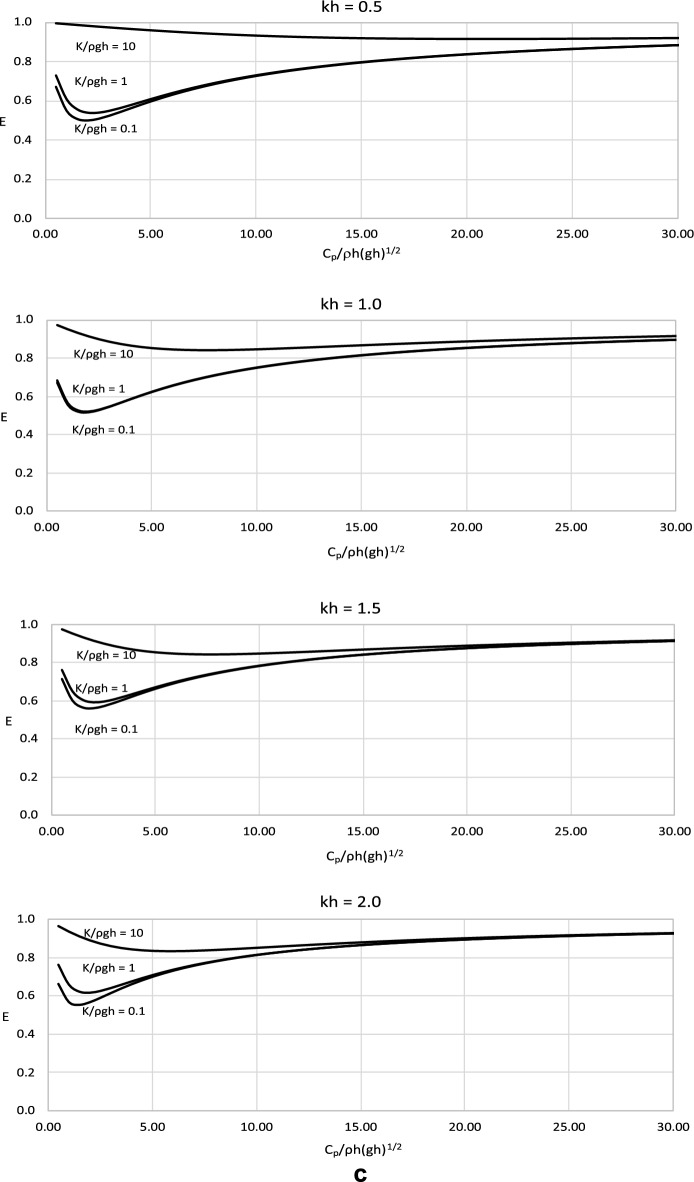
(**a**) Results of *E* versus dimensionless *C*_*p*_/ρ*h*(*gh*)^1/2^ for dimensionless *M*/ρ*h*^2^ = 0.01. (**b**) Results of *E* versus dimensionless *C*_*p*_/ρ*h*(*gh*)^1/2^ for dimensionless *M*/ρ*h*^2^ = 0.1. (**c**) Results of *E* versus dimensionless *C*_*p*_/ρ*h*(*gh*)^1/2^ for dimensionless *M*/ρ*h*^2^ = 1.

The value of *C*_*p*_ and the energy radiated from a converter, *E* =*|R*_*1*_*|*^*2*^ +*|T*_*1*_*|*^*2*^, are the significant factors in the modelling of a wave energy converter. The magnitude of the wave energy radiated by the converter is calculated for a wide range of the parameters of the model. The results are plotted versus the dimensionless value of *C*_*p*_ in Fig. 3 for the wave conditions defined by *kh* = 0.5, 1.0, 1.5 and 2.0 which corresponds with wave periods *T* = 13.20, 7.27, 5.44 and 4.60 s.

The results in Fig. 3 show that the wave energy radiated from the wave energy converter decreases with increasing values of *C*_*p*_ for a small range of lower *C*_*p*_ values and then increases. This is due to the fact that both very low and very high values of power take-off coefficient would stop the energy absorption. The extreme values would either lead to a standstill of the device or a stop in capturing energy. The results clearly show a distinctive minimum in radiated wave energy in a range of low *C*_*p*_ values. For this value, wave reflection and transmission are the lowest. This means that the energy captured by the device is the highest possible, and the value of *C*_*p*_ is optimal for the purpose of energy conversion. To the best of the authors’ knowledge, the optimal value of *C*_*p*_ from a radiated wave energy has been derived with this approach for the first time.

**Figure 4:**
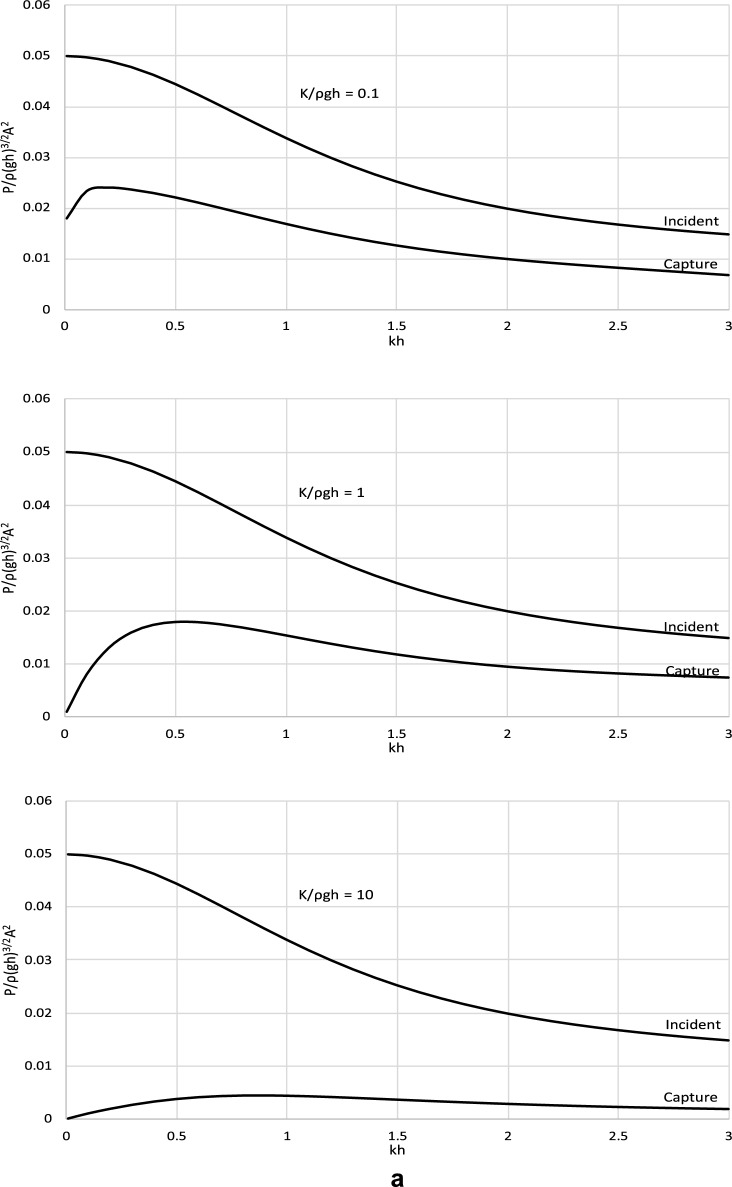

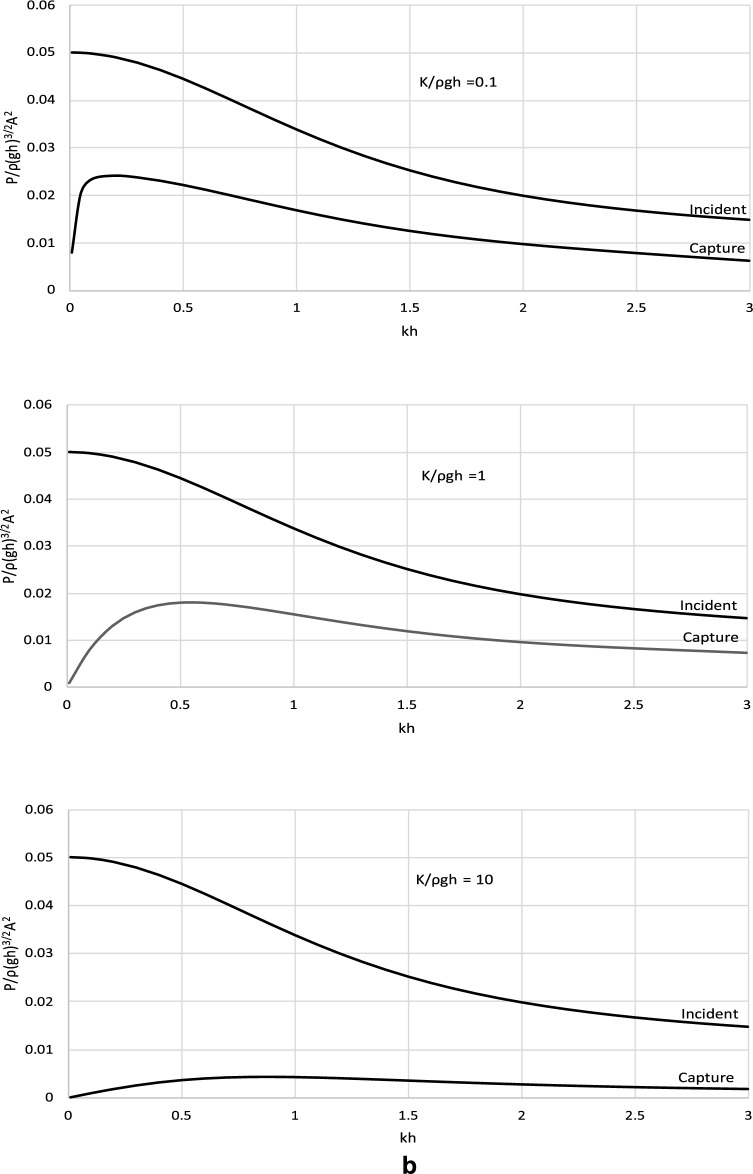

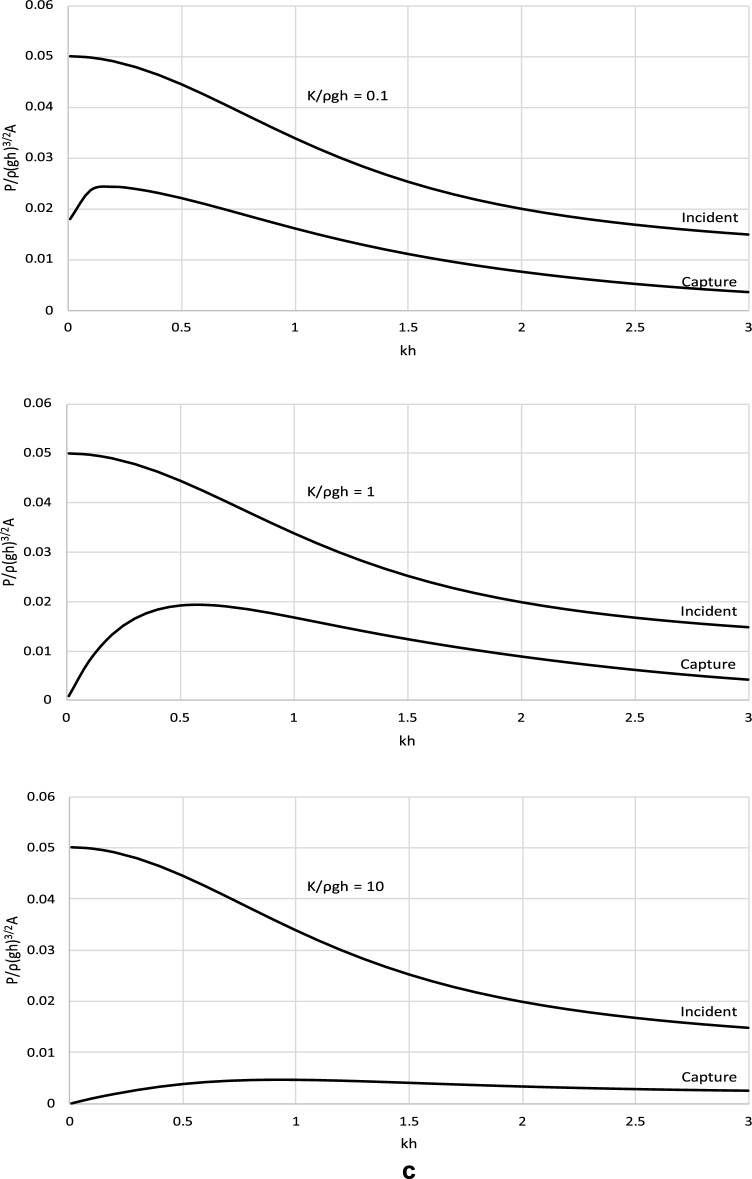
(**a**) Dimensionless results of *P*/ρ(*gh*)^3/2^*A*^2^ versus *kh* for *M*/ρ*h*^2^ = 0.01. (**b**) Dimensionless results of *P*/ρ(*gh*)^3/2^*A*^2^ versus *kh* for *M*/ρ*h*^2^ = 0.1. (**c**) Dimensionless results of *P*/ρ(*gh*)^3/2^*A*^2^ versus *kh* for *M*/ρ*h*^2^ = 1.

**Figure 5:**
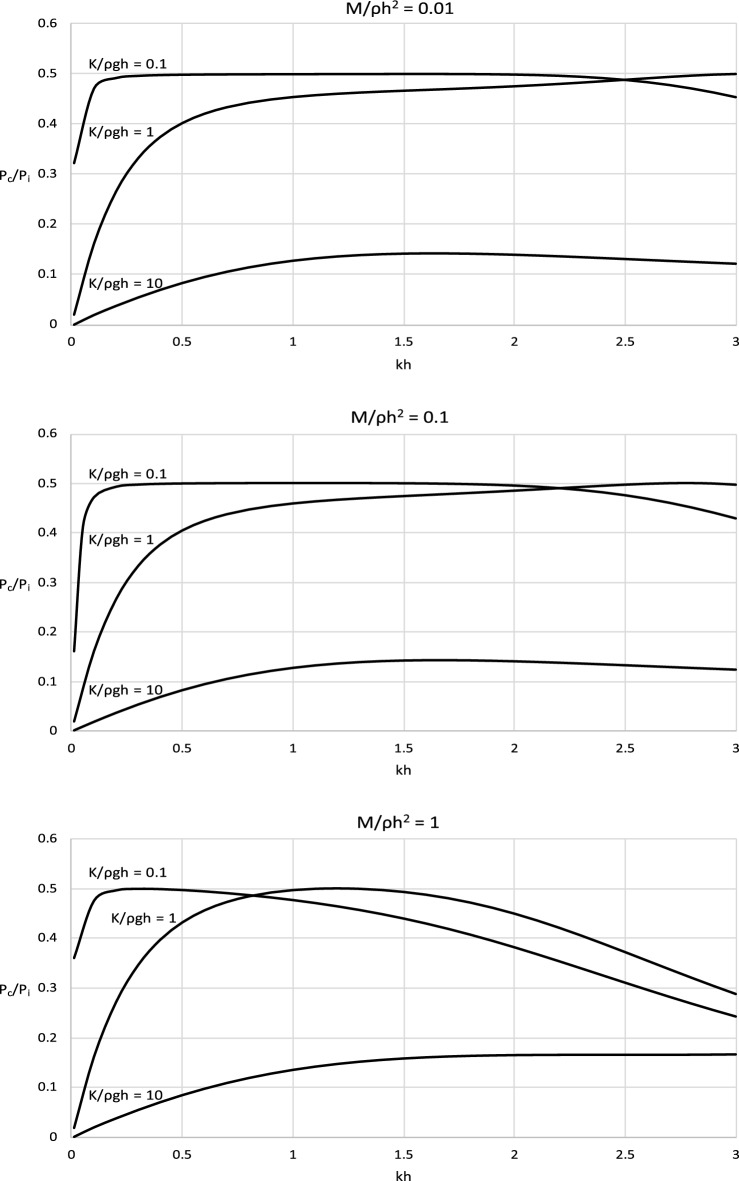
Results of *E* versus dimensionless *C*_*p*_/ρ*h*(*gh*)^1/2^ for dimensionless *M*/ρ*h*^2^ = 0.01.

The derived analytical solution and the formula for the optimal *C*_*p*_ were applied to determine the power efficiency of the wave energy converter operating with such optimal power take-off settings. Figure 4 shows the incident wave power and the power captured by the wave energy converter. Complementary results illustrating the efficiency of a wave energy conversion system are shown in Fig. 5. The results are presented in a dimensionless form and are plotted versus the dimensionless wave number *kh*. The results are presented for representative values of *kh* ∈ (0, 3 > , corresponding to the spectrum of wave periods *T* < *3.67* s, to cover a broad range of wave and water conditions, from shallow to deep waters. The values of stiffness and mass parameters were chosen to comprise typical material parameters as well as extreme values of real engineering material that could be used in the construction processes.

The results in Figs. 4 and 5 show that the wave power captured by the converter increases with increasing wave length until a maximum and then decreases. This is observed in each case basically for the whole spectrum of wave conditions. This indicates that the captured power is very limited for extremely long and short waves. The device is the most effective for waves of moderate lengths for which the captured power is close to optimal. This is a very positive fact from the application viewpoint of the proposed device. The effect of the parameters of the wave energy converter on its efficiency is complex. The results show that the efficiency of the wave energy converter increases with decreasing the stiffness of the system in shallow and intermediate waters. In deep water, the efficiency of the wave energy converter increases with increasing stiffness until a local maximum and then decreases. This is because for this range of wave parameters, by increasing the stiffness of the system radiated wave energy decreases. Moreover, the plots show that the efficiency of the wave energy converter increases with the increasing mass of the system in shallow and intermediate waters. In deep water, the efficiency of the wave energy converter decreases with the increasing mass of the system. This is because for this range of wave parameters, by increasing the mass of the system radiated wave energy decreases.

The derived analytical solution also showed that the top efficiency level of the power capturing mechanism cannot exceed 50%. It is the theoretical maximum for this type of converter for any condition and parameters.

**Figure 6:**
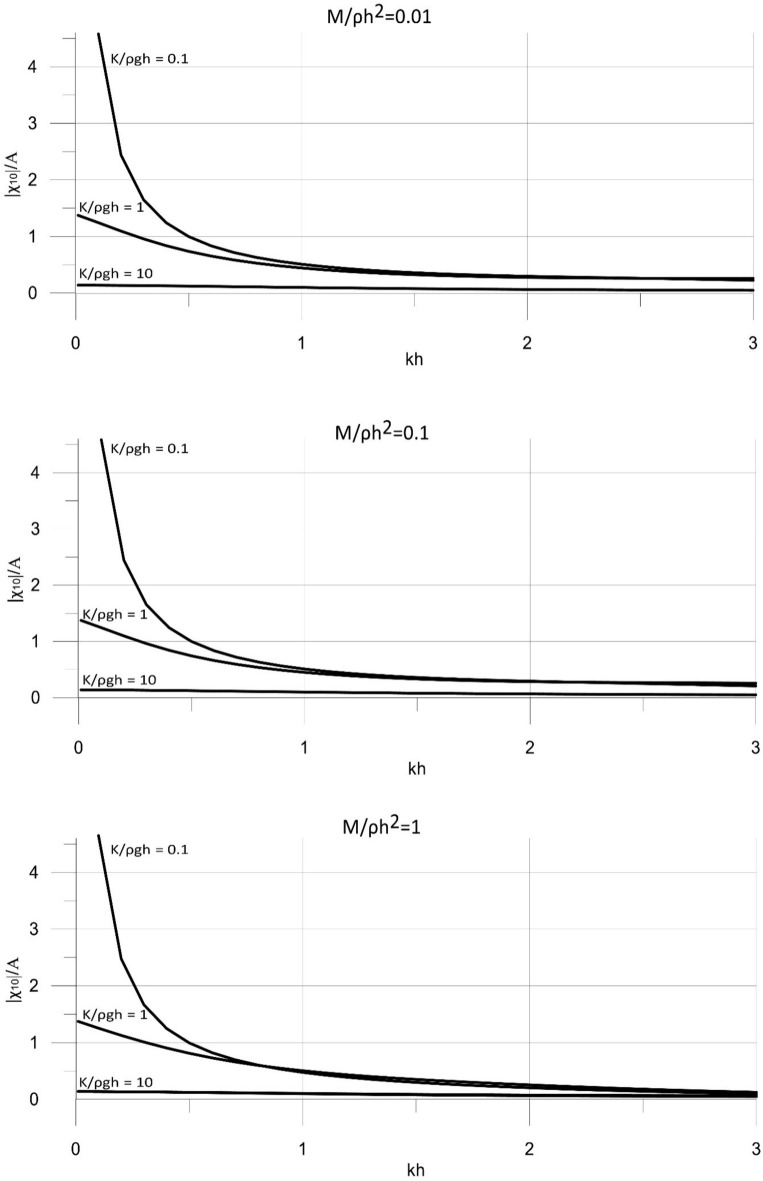
Dimensionless results of |χ_10_| versus *kh* for for *M*/ρ*h*^2^ = 0.01, 0.1, 1.

The model was then applied to determine the displacement of the converter plate. The displacement of the plate was also calculated for the wave energy converter operating with optimal settings for the value of the power take-off coefficient applied in the calculations. The results are shown in Fig. 6 for the main parameters of the model. The results for the displacements of a converter plate are presented in a dimensionless form, |χ_10_|/*A*, and are plotted against the dimensionless wave number, *kh* ∈ (0, 3 > , corresponding to the spectrum of wave periods *T* < 3.67 s, to cover a wide range of wave conditions.

The results in Fig. 6 show that the displacement of the wave energy converter increases with increasing wave length. This is observed for wide ranges of mass and stiffness of the wave energy conversion system. The stiffness of the system has a significant effect on the displacement of the plate. The results show that the displacement decreases with the increasing stiffness of the system. High stiffness values may lead to a standstill, which eventually would stop the conversion of wave energy. A less pronounced effect on the displacement of the converter plate has the mass of the system. The results show that the displacement of the wave energy converter, for a small range of parameters, increases with the increasing mass of the system. This phenomenon is caused by complex nonlinear effects of the parameters of the system on the final results.

**Figure 7:**
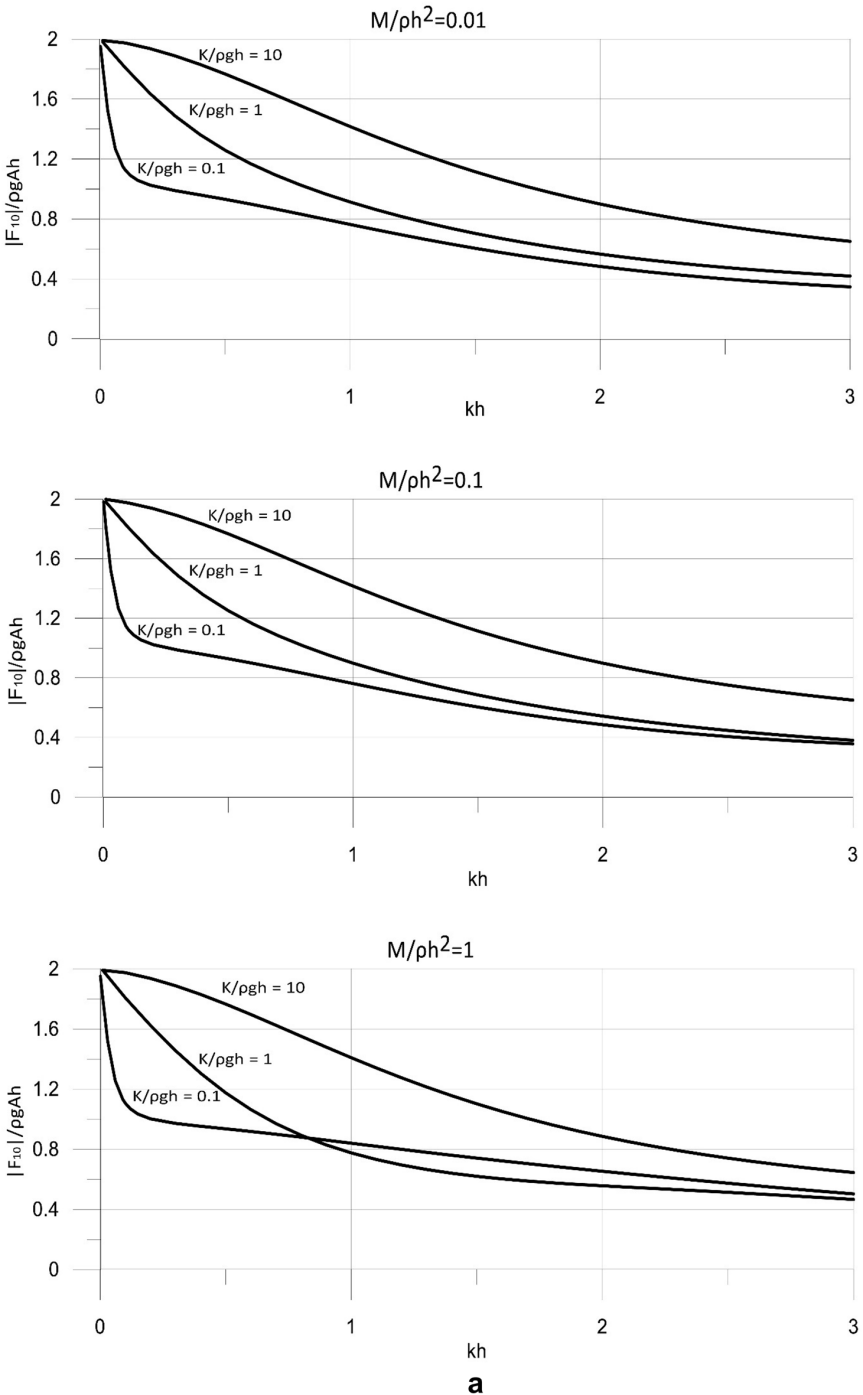

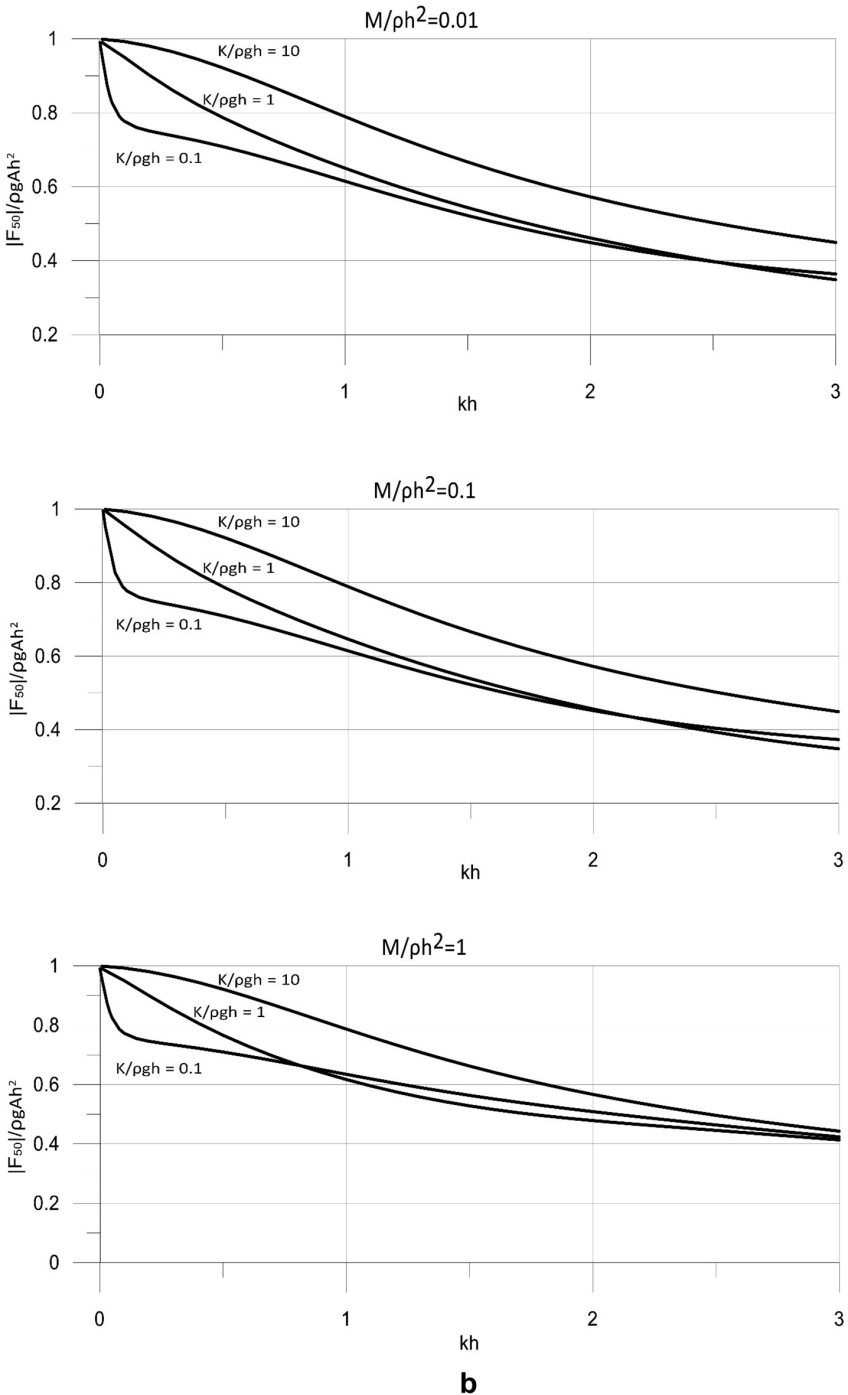
(**a**) Dimensionless results of |*F*_10_| versus *kh* for for *M*/ρ*h*^2^ = 0.01, 0.1, 1. (**b**) Dimensionless results of |*F*_50_| versus *kh* for for *M*/ρ*h*^2^ = 0.01, 0.1, 1.

The derived analytical solution was applied to determine the hydrodynamic wave loads on the wave energy converter. The loads were calculated by integrating the dynamic pressure acting on the working plate of the converter. The calculations were conducted for the wave energy converter operating with the optimal value of the power take-off coefficient. The results are shown in Fig. 7 for the main parameters of the model. The results of the wave load components are presented in a dimensionless form, |*F*_*10*_|/ρ*gAh* and |*F*_*50*_|/ρ*gAh*^*2*^, and are plotted versus the dimensionless wave number *kh* = (0, 3 > and spectrum of wave periods *T* < 3.67 s.

The results in Fig. 7 show that the wave loads acting on the wave energy converter increase with increasing wave lengths. This is observed for wide ranges of mass and stiffness of the wave energy conversion mechanism. The stiffness of the system has a significant effect on the wave loads affecting the working plate. The results show that, in general, the wave loads increase with the increasing stiffness of the system. However, for specific wave conditions, especially for waves of short lengths and in deep waters, the wave loads may decrease with increasing stiffness. This is a non-intuitive result arising from the complex nonlinear effects of the parameters of the system on its efficiency. A less pronounced effect on the loads acting on the converter has the mass of the system. The results show that the wave loads, in general, decrease with increasing the mass of the system, which can be attributed to the nonlinear effects of the parameters of the system on the wave field. However, for specific wave conditions, especially for waves of short lengths and in deep waters, the wave loads may increase while the mass is decreasing.

## Experimental verification

**Figure 8:**
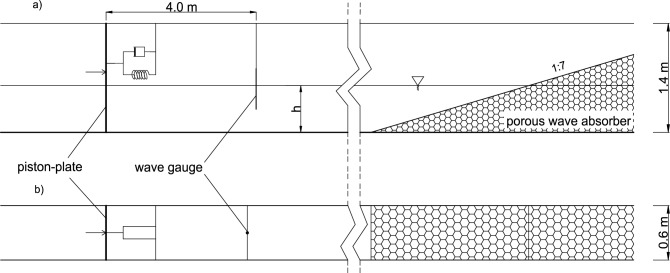
Wave flume setup: (**a**) side view, (**b**) view from above.

Laboratory experiments were conducted in the wave flume of the Institute of Hydro-Engineering of the Polish Academy of Sciences in Gdańsk. The wave flume had 64 m in length, 0.6 m in width and 1.4 m in height. A horizontal steel plate of size 0.6 × 1.4 m was modelling the converter’s oscillations and was installed at one end of the flume. A porous wave absorber of a 1:7 slope was located at the other end of the flume. Wave reflection from the absorber is below 5% for waves considered in typical laboratory experiments^30^. A wave gauge was installed 4 m from the plate system to measure free-surface oscillations. The measurement sampling frequency was 50 Hz. The setup is presented in Fig. 8. Two sets of experiments were carried out for water depths of *h* = 0.6 m and *h* = 0.4 m.

First, preliminary experiments were conducted to establish the accuracy of the measurement system. Then, a set of 5 regular waves of lengths from 1.6 to 7.2 m and wave periods from 2.15 to 5.03 s were generated for each water depth setting. For these parameters, the plate displacement and free-surface oscillations were recorded. Wave parameters and recorded data for each experiment are presented in Table 1.

**Table 1:** Wave parameters and recorded data of all experiments.

Water depth *h* [m]	Experiment no. [–]	Wave length *L* [m]	Wave period *T* [s]	Recorded amplitude *T*_1_ [cm]	Recorded displacement χ_1_ [cm]
0.4	1	1.6	2.15	2.70	1.87
	2	2.4	3.12	2.71	2.65
	3	3.2	4.10	2.83	3.64
	4	4.0	5.10	2.91	4.63
	5	4.8	6.10	2.97	5.69
0.6	1	2.4	1.86	4.09	2.85
	2	3.6	2.63	4.14	4.05
	3	4.8	3.42	4.44	5.68
	4	6.0	4.21	4.39	7.00
	5	7.2	5.03	5.02	9.60

**Figure 9:**
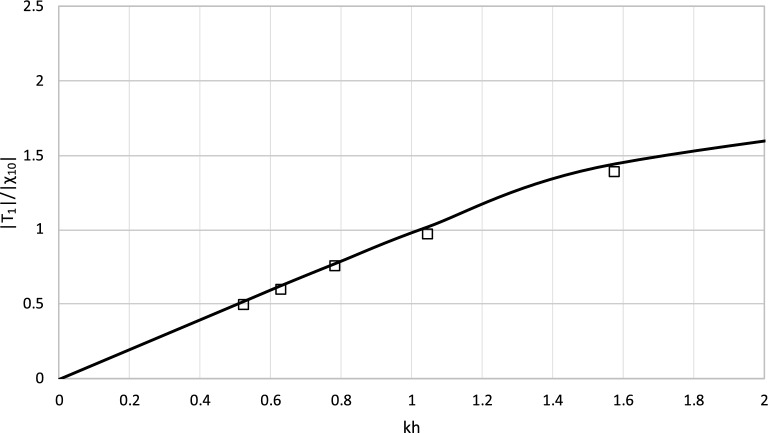
Results of |T_1_| from experiment and model for h = 0.4 m.

**Figure 10:**
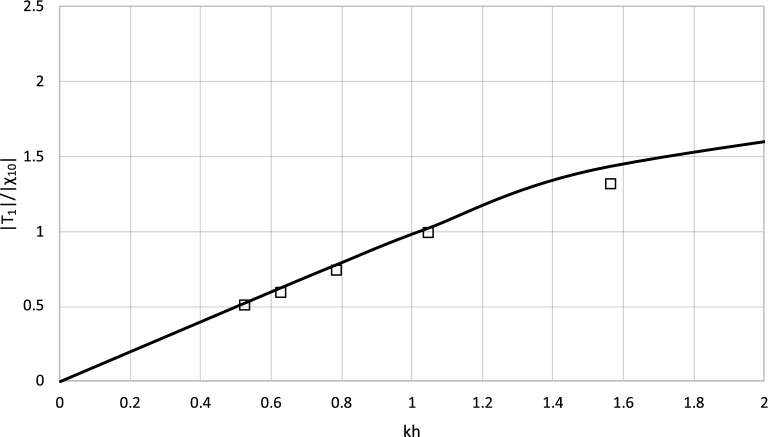
Results of |T_1_| from experiment and model for h = 0.6 m.

To obtain wave parameters, the Fourier analysis was applied. Then, the transmitted wave amplitudes were calculated in the model for the input data from the experiments. The outcome was compared with the experimental results. The transmitted wave amplitudes are presented alongside the regular wave amplitude from the wave flume in Figs. 9 and 10.

The plots illustrate that the theoretical results calculated by the model are in good agreement with the experimental data. The difference between the theoretical results and experimental data is usually below 5%. The discrepancy occurs only for the shortest wave and water depth *h* = 0.6 m and does not recur in the second set of results. It can be concluded that the theoretical model allows to predict the laboratory data with satisfying accuracy.

The radiated waves predicted by the analytical model were compared with experimental results and match waves recorded in laboratory experiments with good accuracies. The validation process chosen in the study relies on the Haskind relations^31^. These relations provide forces from transmitted wave properties. Although it seems to be partial, this type of validation of the model is widely recognized and sufficient from a scientific and an engineering point of view. In fact, the relationship between wave kinematics and wave forces has already been successfully validated in previous works^32^.

## Summary

The problem of wave interaction with a serpent-type wave energy converter was investigated. An original 3D analytical model for wave interaction with a wave energy converter was derived, including the derivation of equations governing the motion of the energy device and the efficiency of a power take-off system. On this theoretical basis, among others, the displacement of the plate of the converter, wave loads on the converter, and the optimal wave energy conversion model were analytically derived.

The analysis shows a strict relation between the basic parameters of the model and the wave energy recoverability. An original analytical formula for the conditions of the optimal operation of the proposed wave energy converter was derived, and a novel analytical formula was obtained to determine the efficiency of a power take-off system. The effect of the values of mass and stiffness of the converter on hydrodynamic forces and converter kinematics were presented.

The results obtained from the analytical formula derived for the efficiency of a wave energy converter are consistent with the original analysis conducted by applying a formula obtained for wave energy. The results show that the wave power captured by the converter increases with increasing wave length until a maximum and then decreases. This is observed basically for the whole spectrum of wave conditions. A more complex effect on the efficiency of a wave energy converter possesses the parameters of a wave energy converter. The results show that the efficiency of a wave energy converter increases with decreasing the stiffness of a system in shallow and intermediate waters. In deep water, the efficiency of a wave energy converter increases with increasing stiffness until a local maximum and then decreases.

Moreover, the results show that the efficiency of a wave energy converter increases with increasing the mass of a system in shallow and intermediate waters. In deep water, the efficiency of a wave energy converter decreases with the increasing mass of a system. The values of the optimal stiffness and mass of the device for given wave conditions were determined. The derived analytical formula shows that the top efficiency level of power capturing cannot exceed 50%. This is the theoretical maximum for this type of converter for any condition and parameters. The power take-off optimization analysis also identifies the spectrum of wave conditions for which the efficiency of the generator is close to the maximum.

Laboratory experiments were conducted in a wave flume to verify the derived analytical model. Radiated waves obtained for different wave lengths and different water depths were compared with theoretical results. A fairly good agreement between the theoretical results and experimental data is observed. According to widely validated and recognized Haskind relations, this outcome verify also forces obtained by the derived model.

The studies indicate that the proposed converter may also be applied for coastal protection by capturing part of wave energy and reducing wave intensity in the coastal zone. Moreover, adding additional functions to the device or using them in highly settings: combining the device with breakwater functions, with wind energy devices or powering desalination plants, fishing farms, or offshore rigs may justify its construction.

## Data Availability

All data used and/or analyzed during the current study are available from the corresponding author on reasonable request.

## Roman symbols

*A*: Amplitude of the incident wave [m]
*C*: Added damping in the equation of motion [Ns/m]
*C*_*p*_: Power take-off coefficient in the equation of motion [Ns/m]
*E*: Coefficient of wave energy [m^2^]
***F***: Generalized hydrodynamic forces [N]
*F*_*1*_: Amplitude of a horizontal force [N]
*F*_*10*_: Spatial amplitude of a horizontal force [N]
*F*_*50*_: Spatial amplitude of a horizontal momentum [Nm]
*F*_*D10*_: Spatial amplitude of the force due to diffracted waves [N]
*g*: Acceleration due to gravity [m/s^2^]
*h*: Water depth [m]
*i*: Imaginary unit [–]
*K*: Stiffness coefficient [N/m]
*k*: Wave number [m^–1^]
*L*: Wave length [m]
*M*: Mass [kg]
***n***: Generalized normal vector [–]
*p*: Wave pressure [Pa]
*P*_*c*_: Wave power captured by the wavemaker [W]
*P*_*I*_: Power of the incident wave [W]
*R*_*1*_: Amplitude of the reflected wave [m]
*R*_*j*_: Amplitude of the evanescent modes associated with the reflected wave and the eigenvalue α_j_ [m]
*T*: Wave period [s]
*T*_*1*_: Amplitude of the transmitted wave [m]
*T*_*j*_: Amplitude of the evanescent modes associated with the transmitted wave and the eigenvalue α_j_ [m]
*t*: Time [s]
***V***: Velocity vector [m/s]
*x*: Principle horizontal coordinate [m]
*y*: Lateral horizontal coordinate [m]
*z*: Vertical coordinate [m]

## Greek symbols

α_j_: Eigenvalue [rad/m]
β_j_: Eigenvalue corresponding to α_j_ [rad/m]
ε: Wave steepness coefficient [–]
θ: Wave propagation angle [rad]
η: Free surface oscillation [m]
μ: Added mass in the equation of motion [kg]
ρ: Fluid density [kg/m^3^]
Φ: Velocity potential [m^2^/s]
ϕ: Spatial velocity potential [m^2^/s]
ϕ_I_: Spatial velocity potential of the incident wave [m^2^/s]
ϕ_1_: Spatial velocity potential of the reflected wave [m^2^/s]
ϕ_2_: Spatial velocity potential of the transmitted wave [m^2^/s]
χ: Displacement of the plate of the converter [m]
χ_1_: Amplitude of the displacement of the plate of the converter [m]
χ_10_: Spatial amplitude of the displacement of the plate of the converter [m]
ω: Wave angular frequency [Hz]
